# Current syphilis infection in virally suppressed people living with HIV: a cross-sectional study in eastern China

**DOI:** 10.3389/fpubh.2024.1366795

**Published:** 2024-06-19

**Authors:** Lin He, Xiaohong Pan, Jiezhe Yang, Jinlei Zheng, Mingyu Luo, Wei Cheng, Chengliang Chai

**Affiliations:** Zhejiang Provincial Center for Disease Control and Prevention, Hangzhou, Zhejiang, China

**Keywords:** antiretroviral therapy, human immunodeficiency virus (HIV), people living with HIV, viral load, syphilis

## Abstract

**Background:**

Antiretroviral therapy (ART) has been shown to reduce human immunodeficiency virus (HIV) viral replication and ultimately achieve viral suppression and eliminate HIV transmission. However, little is known about the impact of viral suppression on high-risk behaviors and sexually transmitted infections (STIs).

**Objective:**

This study aimed to assess the rates of current syphilis infection in virally suppressed people living with HIV (PLWH) and whether with the duration of ART can reduce the current syphilis infection in eastern China.

**Method:**

We conducted a cross-sectional survey of PLWH in Zhejiang Province, China, in 2022. PLWH who were on ART >6 months and were virally suppressed (viral load <50 copies/mL) were included in the study. Data were collected from the National Epidemiological Database of Zhejiang Province and all participants were tested for viral load and current syphilis. Multivariable logistic regression was used to identify risk factors associated with current syphilis infection.

**Result:**

A total of 30,744 participants were included in the analysis. 82.7% of participants were male, the mean age was 44.9 ± 14.1 years, 84.9% had received ART in a hospital setting, the mean time on ART was 5.9 ± 3.1 years and 5.6% of participants were infected with current syphilis. Multivariable logistic regression showed that being male [adjusted odds ratio (aOR): 2.12, 95% confidence interval (CI): 1.69–2.66], high level of education (aOR: 1.23, 95% CI: 1.02–1.49), homosexual route of HIV infection (aOR: 1.80, 95% CI: 1.60–2.04), non-local registered residence (aOR: 1.29, 95% CI: 1.11–1.51), had history of STIs before HIV diagnosis (aOR: 1.95, 95 % CI: 1.75–2.18) and treatment provided by a municipal hospital (aOR: 2.16, 95% CI: 1.31–3.55) were associated with increased risk of current syphilis infection. Being married (aOR: 0.67, 95% CI: 0.58–0.76) was associated with a decreased risk of current syphilis infection.

**Conclusion:**

Our findings revealed a high rate of current syphilis infection among virally suppressed PLWH in eastern China. Duration of ART did not reduce the prevalence of current syphilis infection. Targeted interventions to reduce current syphilis infection should be prioritized for subgroups at higher risk.

## Key messages

1. Our findings revealed 5.6% of current syphilis infection among HIV-infected patients with successfully suppressed viral load in eastern China.

2. Antiretroviral therapy does not reduce the rate of high-risk sexual behavior and does not prevent the current infection with syphilis. PLWH who are male, high level of education, homosexual, non-local residence, had history of STIs and from municipal hospital are at higher risk of syphilis infection.

3. Targeted interventions should be implemented to reduce high-risk sexual behavior and STIs infection.

## Background

The advent of antiretroviral therapy (ART) has changed the landscape of the prevention and treatment of human immunodeficiency virus (HIV) infection. Early initiation of ART is effective in reducing morbidity and mortality, as well as ongoing transmission of the disease (1–3). Based on the growing body of high-quality literature supporting this treatment as a preventive approach (4), the Joint United Nations Program on HIV/AIDS (UNAIDS) proposed the 95-95-95 targets. The overall goal of this strategy is to achieve 95% diagnosis of HIV-infected individuals, 95% treatment of those diagnosed, and 95% viral suppression of those treated, to reduce transmission and ultimately end the global HIV epidemic by 2030 (5, 6). A recent European study of 1,000 HIV-serodiscordant male couples found that over an 8-year period, there were no cases of the HIV-negative partner contracting the virus through unprotected sex (7). These findings support the UNAIDS strategy of “undetectable equals untransmittable” (“U=U”) (8, 9), stating that people living with HIV (PLWH) on ART who have an undetectable levels of HIV in their blood have a negligible risk of sexually transmitting HIV. These findings continue to underscore the importance of early initiation of ART in reducing the number of new infections and its role in contributing to end of the HIV pandemic.

U=U means that PLWH who achieve and maintain sustained viral load (VL) suppression to an undetectable VL for at least 6 months, and who take ART medications as prescribed, do not transmit HIV to sexual partners. Several challenges remain to achieving U=U despite the implementation of the U=U strategy to reduce HIV transmission. First, while ART can reduce the transmission of HIV, it does not reduce the incidence of other sexually transmitted infections (STIs) (7). Owing to the benefits of ART, patients' physical health of patients improves after starting treatment, and some studies have reported that these individuals resume high-risk sexual behaviors (10). Some PLWH with a suppressed VL may no longer consider themselves to be infectious and may no longer use a condom when they engage in sexual activity, and syphilis will be a reemerging disease among PLWH. A previous study showed that 44.6% of PLWH with viral suppression had syphilis (11), indicating a significant risk of STIs despite HIV viral suppression in their blood plasma. In addition, the presence of STIs and genital tract inflammation are associated with higher infectiousness, even when the virus is suppressed (12). Second, HIV is a chronic disease that requires lifelong therapy. Drug toxicity or HIV drug resistance can develop in patients with poor compliance or adherence to antiviral therapy (ART) (13), problems which can lead to treatment failure, resulting in high VL rebound and HIV transmission (14). Third, in many countries, including China, HIV VL testing is only performed once a year in patients receiving ART. With such long intervals between HIV VL testing, patients may experience fluctuations in their VL levels, particularly if they are do not adherent to ART. These problems can lead not only to new HIV infections, but also to new infection with another quasispecies or subtype of HIV in casual encounters with HIV-positive partners, and can occur, and reverse the success of ART programs when combined with the potential for high-risk sexual behaviors. Patients with viral suppression and STIs should be targeted for interventions to reduce risky behaviors and HIV VL testing should be performed more frequently to monitor their viral suppression levels.

HIV and Syphilis co-infection has been on the rise in recent years (15). Syphilis and HIV are sexually transmitted, condomless and multiple sexual partner behaviors increase the risk of HIV and syphilis infection. A retrospective cohort study showed that syphilis not only increased HIV, but also increased Mpox (16). Since May, 2022, ~90,000 Mpox infections have been diagnosed in 116 countries, primarily transmitted through sexual activity among men who have sex with men (MSM) (17). HIV-infected patients account for 38–50% of people diagnosed with Mpox (18–20). A study of the Mpox outbreak in several countries showed that about 65% of PLWH were adherent to ART (21). In the Southeastern U.S, the study found that among PLWH with a known HIV viral load, 65% had a ≤ 200 copies/mL (22). Most of the PLWH described in the 2022 Mpox case series were HIV viral suppressed (<200 copies/mL) (23). A study showed that 50% of Mpox patients had syphilis (24). These studies suggested that sexually active behavior still exists among HIV-infected people who had HIV viral suppression, patients who achieve undetectable HIV VL still had risk for infect Mpox and STIs.

With the implementation of U=U strategy, more and more patients achieve undetectable HIV VL, it is also widely recognized that high-risk behaviors can continue at any time after HIV diagnosis, what is the STIs status of these patients? STIs infection can be used to scientifically assess the sexual behavior of PLWH, and HIV viral suppression and ART should be considered in the broader context of risky sexual behavior and sexual health. However, few studies have focused on the assessment of high-risk sexual behaviors and STIs in virally suppressed PLWH, and no such studies have been conducted in China. We aimed to assess the rates and associated risk factors of current syphilis infection among PLWH with viral suppression achieving viral suppression in Eastern China, where more than 90% of PLWH are receiving ART and are virally suppressed, which is of great importance for the prevention and control of HIV, Mpox and STIs transmission.

## Methods

### The ART program in China

In China, after patients have diagnosed with HIV, the local Center for Disease Control and Prevention (CDC) or other health care institutions, such as hospitals or community health service centers, provide follow-up care once or twice a year. To reduce HIV transmission, this care includes interventions targeting high-risk sexual behaviors. In addition, China's free National ART Program provides free ART to all PLWH. Approximately 77% of PLWH in China start ART within 1 month of HIV diagnosis in 2023. Initially, the local CDC was the sole distributor of ART. However, over the past decade, there has been a decentralization of HIV care to local hospitals (25). All patients on ART receive follow-up care every 3 months from these local treatment institutions.

### Study design

At the end of December 31, 2022, all PLWH residing in Zhejiang Province in eastern China were identified and a cross-sectional survey was conducted. Data on PLWH were obtained from the Zhejiang Provincial National Epidemiological Database, which tracks all persons diagnosed with HIV in China. Zhejiang is an economically prosperous, southeastern coastal province with more than 39,744 PLWH at the end of 2022, ranking ninth among all 31 provinces in China in terms of the number of diagnosed PLWH (unpublished surveillance data). According to recent surveillance data, 85.7% of PLWH have been diagnosed, 95.0% of diagnosed patients are on ART, and 94.4% of the patients on ART are virally suppressed (defined as VL < 50 copies/mL) (26). All known PLWH living in Zhejiang Province before December 31, 2022, were enrolled in this study.

### Participants

The inclusion criteria were as follows: (1) age ≥ 15 years; (2) on ART > 6 months; (3) VL < 50 copies/mL. The Institutional Review Board of the Zhejiang Provincial Center for Disease Control and Prevention approved this study. All participants provided written informed consent before completing the questionnaire. Syphilis testing was free for all participants, and investigators were required to inform participants of their results. The personal information of the study participants was kept confidential.

### Data collection

Participants were identified from the National Epidemiological Database of Zhejiang Province, which tracks all diagnosed cases in the province. The database included the date of HIV diagnosis, level of education, HIV transmission route and other social demographic characteristics. Patients receiving ART received regular follow-up care, which mainly included clinical evaluation and laboratory monitoring, such as CD4 testing, routine blood tests, liver, assessment of kidney function, VL and treatment information. The local treatment institutions complete a questionnaire after each follow-up visit.

### Syphilis rapid plasma reagin testing

The presence of syphilis is an indication of a history of high-risk sexual behavior (27). A 5-milliliter (mL) venous blood specimen was drawn from each participant following the interview. First, syphilis seropositivity testing was performed using the Treponema pallidum particle agglutination assay (*TPPA*). Second, syphilis *TPPA* positivity testing was performed using rapid plasma reagin (*RPR*) to confirm Treponema pallidum syphilis infection. In this study, all participants underwent testing for syphilis between March and December 2022.

### Viral load testing

VL testing is offered free of charge every 12 months in China. Therefore, VL data from individuals tested between January and September 2022 were used in this study. A quantitative polymerase chain reaction (*PCR*) technique was used to measure VL, with a lower limit of detection of 50 copies/mL. In this study, the lower limit of detection (<50 copies/mL) was defined as viral suppression.

### Statistical analysis

Data were double entered into an EpiData version 3.1 database. The statistical analysis was performed using SPSS version 19.0 (IBM Corp. Released 2010. IBM SPSS Statistics for Windows, Version 19.0. Armonk, NY: IBM Corp.). For descriptive analyses, categorical variables were reported as frequencies and proportions, and continuous variables were reported as medians and interquartile ranges (IQR) or means and standard deviations (SDs). Differences in demographic characteristics and sexual risk behaviors were calculated using a Chi-squared (χ2) test. Factors from the univariable logistic regression with *P* < 0.10 and/or factors from previous studies shown to be associated with STIs were included in multivariable logistic regression models, and adjusted odds ratios (aORs) were calculated along with 95% confidence intervals (CIs). The statistical significance level was set at *P* ≤ 0.05 and β = 0.1.

### Ethics considerations

The Institutional Review Board of the Zhejiang Provincial Center for Disease Control and Prevention approved this study. All participants provided written informed consent prior to completion of the survey. Syphilis testing and viral load testing were free for all participants, and the investigators were required to tell HIV-infected individuals their results. The personal information of the study participants was kept confidential.

## Results

**Figure 1:**
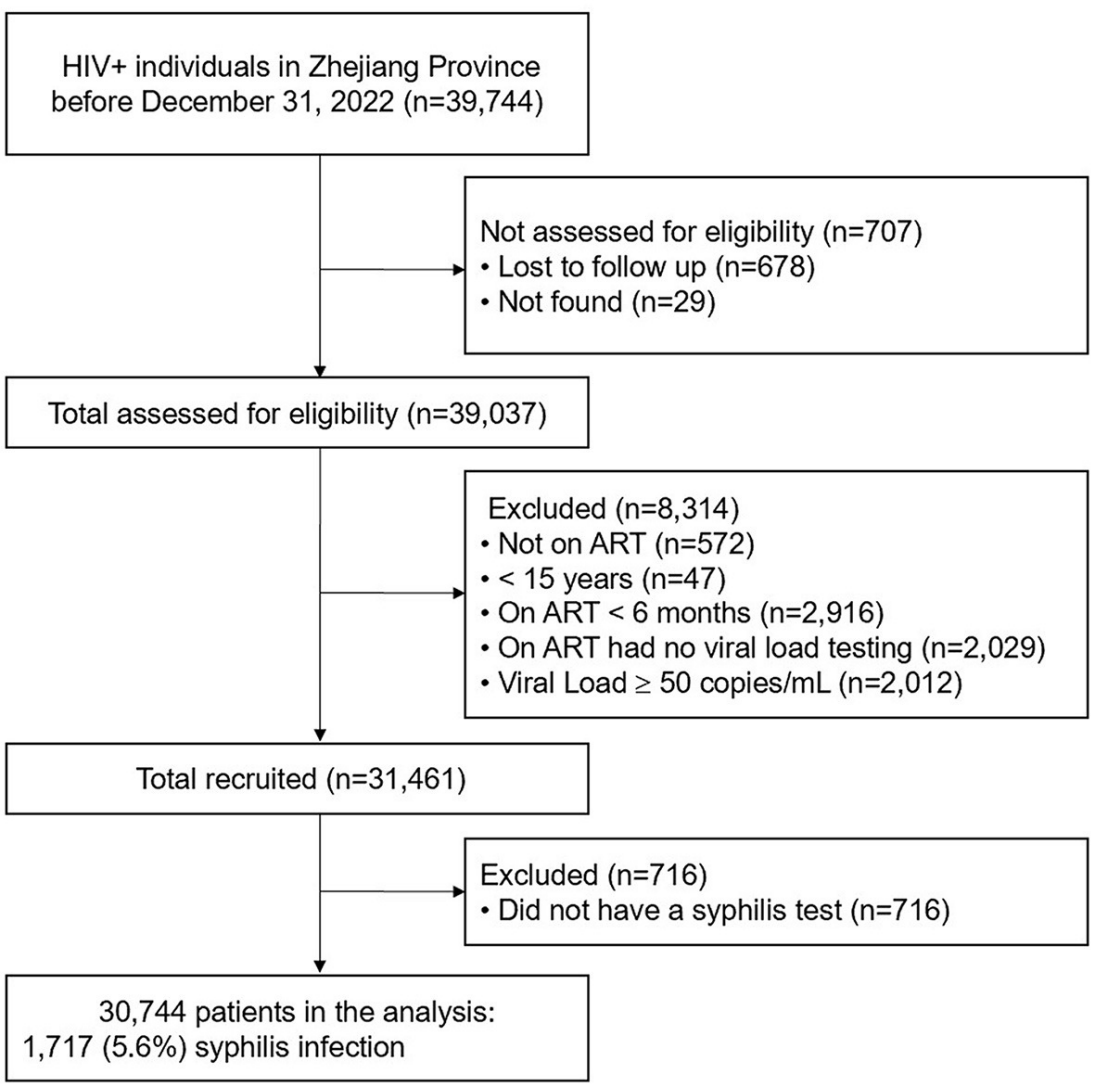
Study design.

The study enrollment process is shown in Figure 1. Of the 39,744 PLWH living in Zhejiang Province at the end of December 2022, 30,744 met the inclusion criteria and were included in the final study. Of the 30,744 participants, 82.7% were male, the mean age was 44.9 ± 14.1 years; and 54.1 and 44.7% were reported to have acquired HIV infection through heterosexual and homosexual intercourse, respectively. 18.7% of patients had a history of STIs before their HIV was diagnosis and 84.9% had received ART in a hospital setting. The mean CD4 count was 516 ± 238 cells/μL, the mean time on ART was 5.9 ± 3.1 years, and the longest time on ART was 18.8 years (Table 1).

**Table 1:** Demographic characteristics and sexual risk behaviors of participants with and without syphilis.

**Variables**	***N* (Column %)**	**Negative *n* (%)**	**Positive *n* (%)**	**χ2**	***P***
Overall	30,744 (100)	29,027 (94.4)	1,717 (5.6)		
**Sex**				178.951	<0.001
Female	5,311 (17.3)	5,218 (98.2)	93 (1.8)		
Male	25,433 (82.7)	23,809 (93.6)	1,624 (6.4)		
**Age (years)**				179.158	<0.001
**IQR 43 (33–55)**					
16–24	1,008 (3.3)	936 (92. 9)	72 (7.1)		
25–34	7,758 (25.2)	7,181 (92.6)	577 (7.4)		
35–44	7,452 (24.2)	6,966 (93.5)	486 (6.5)		
45–54	6,605 (21.5)	6,250 (94.6)	355 (5.4)		
55–	7,921 (25.8)	7,694 (97.1)	227 (2.9)		
**Marital status**				200.201	<0.001
Single	12,166 (39.6)	11,236 (92.4)	930 (7.6)		
Married	12,894 (41.9)	12,437 (96.5)	457 (3.5)		
Divorced/separated	5,684 (18.5)	5,354 (94.2)	330 (5.8)		
**Ethnicity**				0.039	0.844
Minority	992 (3.2)	938 (94.6)	54 (5.4)		
Han	29,752 (96.8)	28,089 (94.4)	1,663 (5.6)		
**Education**				130.247	<0.001
Primary school/illiteracy	6,924 (22.5)	6,716 (97.0)	208 (3.0)		
Junior high school	10,514 (34.2)	9,917 (94.3)	597 (5.7)		
High school and junior college	5,930 (19.3)	5,542 (93.5)	388 (6.5)		
College	7,376 (24.0)	6,852 (92.9)	524 (7.1)		
**Route of HIV infection**				367.791	<0.001
Heterosexual	16,631 (54.1)	16,074 (96.7)	557 (3.3)		
Homosexual	13,741 (44.7)	12,590 (91.6)	1,154 (8.4)		
Others	372 (1.2)	363 (97.6)	9 (2.4)		
**Registered residence**				11.468	0.003
Local	25,623 (83.3)	24,221 (94.5)	1,402 (5.5)		
Other city of Zhejiang	2,370 (7.7)	2,247 (94.8)	123 (5.2)		
Other province	2,751 (8.9)	2,559 (93.0)	192 (7.0)		
**History of STIs before HIV diagnosis**				210.383	<0.001
No	21,718 (70.6)	20,709 (95.4)	1,009 (4.6)		
Yes	5,754 (18.7)	5,205 (90.5)	549 (9.5)		
Unknown	3,272 (10.6)	3,113 (95.1)	159 (4.9)		
**Treatment institutions**				33.365	<0.001
CDC	4,147 (13.5)	3,915 (94.5)	230 (5.5)		
Municipal hospital	7,821 (25.4)	7,289 (93.2)	532 (6.8)		
County hospital	18,285 (59.5)	17,347 (94.9)	938 (5.1)		
Community health service center	491 (1.6)	474 (96.5)	17 (3.5)		
**CD4 count (cells/μL)**				4.764	0.092
**IQR 487 (347–651)**					
<200	1,992 (6.5)	1,898 (95.3)	94 (4.7)		
200–499	14,037 (45.7)	13,219 (94.2)	818 (5.8)		
500–	14,715 (47.9)	13,910 (94.5)	805 (5.5)		
**Duration of ART (years)**				179.951	<0.001
**IQR 5.5 (3.4–8.0)**					
<2	3,309 (10.8)	3,140 (94.9)	169 (5.1)		
2-	6,520 (21.2)	6,157 (94.4)	363 (5.6)		
4-	6,941 (22.6)	6,528 (94.0)	413 (6.0)		
6-	6,402 (20.8)	6,047 (94.5)	355 (5.5)		
8-	4,243 (13.8)	3,985 (93.9)	258 (6.1)		
10-	3,329 (10.8)	3,170 (95.2)	159 (4.8)		

IQR, interquartile range; HIV, human immunodeficiency virus; STIs, sexually transmitted infections; CDC, Center for Disease Control; ART, anti-retroviral therapy.

Of the 30,744 participants 1,717 (5.6%) had current syphilis infection. Of those who tested positive for syphilis, 94.6% were men, 37.8% of participants were aged 25–34 years, and 54.2% were single. Of the participants with current syphilis infection, 67.2% reported being infected through homosexual transmission, and 32.0% had no history of STIs before their HIV diagnosis. Of the participants who tested positive for syphilis, 53.1% had CD4 counts of ≤ 499 cells/μL. The χ2 test showed that all factors, except for ethnicity and CD4 count differed significantly between participants with and without syphilis (Table 1).

The results of the multivariable logistic analysis are summarized in Table 2. The duration of ART was not associated with the syphilis in the multivariable model. Participants who were male (aOR: 2.12, 95% CI: 1.69–2.66), had high level of education (aOR: 1.23, 95% CI: 1.02–1.49), had acquired HIV through homosexual intercourse (aOR: 1.80, 95% CI: 1.60–2.04), with a non-local registered residence (aOR: 1.29, 95% CI: 1.11–1.51), with a history of STIs before the HIV diagnosis (aOR: 1.95, 95 % CI: 1.75–2.18), and treatment provided at a municipal hospital (aOR: 2.16, 95% CI: 1.31–3.55) were associated with increased risk of syphilis. Being married (aOR: 0.67, 95% CI: 0.58–0.76) was associated with a decreased risk of syphilis (Table 2).

**Table 2:** Risk factors for current syphilis infection in virally suppressed people living with HIV.

**Variables**	**Current syphilis infection**		
	**% (** ***n*** **/** ***N*** **)**	**OR 95% CI**	**aOR 95% CI**
Overall	5.6 (1,717/30,744)		
**Sex**			
Female	1.8 (93/5,311)	1.00	1.00
Male	6.4 (1,624/25,433)	3.83 (3.10–4.73)	2.12 (1.69–2.66)
**Marital status**			
Single	7.6 (930/12,166)	1.00	1.00
Married	3.5 (457/12,894)	0.44 (0.40–0.50)	0.67 (0.58–0.76)
Divorced/separated	5.8 (330/5,684)	0.75 (0.65–0.85)	0.99 (0.86–1.14)
**Education**			
Primary school/illiteracy	3.0 (208/6,924)	1.00	1.00
Junior high school	5.7 (597/10,514)	1.94 (1.66–2.28)	1.37 (1.16–1.62)
High school and junior college	6.5 (388/5,930)	2.26 (1.90–2.69)	1.23 (1.02–1.49)
College	7.1 (524/7,376)	2.47 (2.10–2.91)	1.13 (0.94–1.37)
**Route of HIV infection**			
Heterosexual	3.3 (557/16,631)	1.00	1.00
Homosexual	8.4 (1,151/13,741)	2.64 (2.38–2.93)	1.80 (1.60–2.04)
Others	2.4 (9/372)	0.72 (0.37–1.39)	0.76 (0.39–1.49)
**Registered residence**			
Local	5.5 (1,402/25,623)	1.00	1.00
Other city of Zhejiang	5.2 (123/2,370)	0.95 (0.78–1.14)	0.90 (0.75–1.10)
Other province	7.0 (192/2,751)	1.30 (1.11–1.52)	1.29 (1.11–1.51)
**History of STIs before HIV diagnosis**			
No	4.6 (1,009/21,718)	1.00	1.00
Yes	9.5 (549/5,754)	2.17 (1.94–2.41)	1.95 (1.75–2.18)
Unknown	4.9 (159/3,272)	1.05 (0.88–1.24)	1.17 (0.98–1.39)
**Treatment institutions**			
CDC	5.5 (230/4,147)	1.64 (0.99–2.70)	2.01 (1.21–3.33)
Municipal hospital	6.8 (532/7,821)	2.04 (1.25–3.33)	2.16 (1.31–3.55)
County hospital	5.1 (938/18,285)	1.51 (0.93–2.46)	1.93 (1.18–3.16)
Community health service center	3.5 (17/491)	1.00	1.00

aOR, adjusted odds ratio; CDC, Centers for Disease Control; CI, confidence interval; HIV, human immunodeficiency virus; OR, odds ratio; STIs, sexually transmitted infection.

The prevalence of current syphilis was 3.3% (557/16,331) in heterosexual participants, and 8.4% (1,151/13,741) in homosexual participants. The results of the multivariable logistic regression analysis among heterosexual and homosexual participants are summarized in Table 3. In heterosexual participants, being male (aOR: 1.87, 95% CI: 1.49–2.36), having high level of education (aOR: 1.46, 95% CI: 1.10–1.96), history of STIs before the HIV diagnosis (aOR: 2.47, 95% CI: 2.04–2.99), and treatment provided at municipal (aOR: 5.47, 95% CI: 1.34–17.03) were associated with higher risk of syphilis. Being married (aOR: 0.50, 95% CI: 0.40–0.63) was associated with lower risk of syphilis. In participants with homosexually acquired HIV infection, having junior high school (aOR: 1.33, 95% CI: 1.01–1.74), non-local registered residence (aOR: 1.32, 95% CI: 1.09–1.60), and history of STIs before HIV diagnosis (aOR: 1.73, 95% CI: 1.51–1.97) were associated with an increased risk of syphilis. Being married (aOR: 0.76, 95% CI: 0.64–0.90) was associated with lower risk of syphilis (Table 3).

**Table 3:** Risk factors for current syphilis infection in study participants according to their mode of acquiring HIV infection.

**Variables**	**Current syphilis in heterosexual cases**			**Current syphilis in homosexual cases**		
	**% (** ***n*** **/** ***N*** **)**	**OR 95% CI**	**aOR 95% CI**	**% (** ***n*** **/** ***N*** **)**	**OR 95% CI**	**aOR 95% CI**
Overall	3.3 (557/16,631)			8.4 (1,151/13,741)		
**Sex**				–	–	–
Female	1.8 (93/5,179)	1.00	1.00	–	–	–
Male	4.1 (464/11,452)	2.31 (1.84–2.89)	1.87 (1.49–2.36)	–	–	–
**Marital status**						
Single	5.4 (182/3,364)	1.00	1.00	8.6 (743/8,679)	1.00	1.00
Married	4.0 (233/9,643)	0.43 (0.36–0.53)	0.55 (0.44–0.68)	7.2 (171/3,073)	0.83 (0.71–0.97)	0.76 (0.64–0.90)
Divorced/separated	3.9 (142/3,624)	0.71 (0.57–0.89)	0.88 (0.69–1.12)	9.4 (187/1,989)	1.11 (0.94–1.31)	1.00 (0.84–1.20)
**Education**						
Primary school/illiteracy	2.3 (136/5,811)	1.00	1.00	7.1 (69/975)	1.00	1.00
Junior high school	3.4 (221/6,449)	1.48 (1.19–1.84)	1.29 (1.03–1.61)	9.6 (374/3,903)	1.39 (1.07–1.82)	1.33 (1.01–1.74)
High school and junior college	4.1 (101/2,477)	1.77 (1.37–2.31)	1.32 (1.00–1.74)	8.4 (287/3,415)	1.21 (0.92–1.58)	1.11 (0.84–1.47)
College	5.2 (99/1,894)	2.30 (1.77–3.00)	1.46 (1.10–1.96)	7.7 (421/5,448)	1.10 (0.84–1.43)	1.00 (0.75–1.32)
**Registered residence**						
Local	3.4 (473/14,097)	1.00	–	8.2 (924/11,289)	1.00	1.00
Other city of Zhejiang	2.5 (29/1,174)	0.73 (0.50–1.07)	–	8.1 (93/1,145)	0.99 (0.79–1.24)	0.97 (0.78–1.21)
Other province	4.0 (55/1,360)	1.21 (0.91–1.61)	–	10.3 (134/1,307)	1.28 (1.06–1.55)	1.32 (1.09–1.60)
**History of STIs before HIV diagnosis**						
No	2.7 (322/11,956)	1.00	1.00	7.2 (682/9,517)	1.00	1.00
Yes	6.9 (176/2,544)	2.69 (2.22–3.24)	2.47 (2.04–2.99)	11.7 (373/3,185)	1.72 (1.50–1.96)	1.73 (1.51–1.97)
Unknown	2.8 (59/2,131)	1.03 (0.78–1.36)	1.04 (0.79–1.38)	9.2 (96/1,039)	1.32 (1.05–1.65)	1.27 (1.02–1.59)
**Treatment institutions**						
CDC	3.8 (89/2,323)	3.94 (0.96–16.14)	4.99 (1.22–20.48)	7.9 (139/1,767)	1.34 (0.77–2.32)	–
Municipal hospital	4.4 (143/3,277)	4.52 (1.11–18.37)	5.47 (1.34–22.32)	8.7 (387/4,473)	1.48 (0.87–2.53)	–
County hospital	3.0 (323/10,831)	3.04 (0.75–12.31)	4.20 (1.04–17.03)	8.4 (610/7,251)	1.44 (0.85–2.44)	–
Community health service center	1.0 (2/200)	1.00	1.00	6.0 (15/250)	1.00	–

aOR, adjusted odds ratio; CDC, Centers for Disease Control; CI, confidence interval; HIV, human immunodeficiency virus; OR, odds ratio; STI, sexually transmitted infection.

## Discussion

This study assessed the prevalence of current syphilis infection among virally suppressed PLWH in Zhejiang Province, China, in 2022. According to national reporting data, the annual incidence of primary and secondary syphilis have decreased from 13.44% in 2012 to 9.81% in 2015 (28), and the overall prevalence of syphilis among PLWH in China was 19.9% (29). Furthermore, according to 2011 global statistics, the mean prevalence of syphilis among PLWH was 9.5% (30). Of the ~30,744 participants in this study, 5.6% tested positive for syphilis infection, a study from the USA showed that 34% of HIV men diagnosed with syphilis had a repeat syphilis diagnosis within 3 years (31), and a study from an HIV clinic showed that 11.1–24.4% of PLWH had syphilis reinfection (32), suggesting that syphilis remains a public health challenge, especially among PLWH, and that patients who are virally suppressed should still use condoms when having sexual intercourse outside the context of a mutually monogamous relationship. In China, syphilis is the most commonly reported STIs and consistently ranks among the top five reported communicable diseases (33). In the presence of HIV, STIs such as syphilis can lead to increased infectiousness (12), thwarting the progress made through adherence to ART regimens and potentially leading to increased HIV transmission. Efforts to identify those at increased risk of STIs, especially among virally suppressed individuals, are needed if eastern China is to maintain its high prevalence of viral suppression in line with the 95-95-95 goals.

Several studies have reported an increasing in the incidence of syphilis epidemics in certain key groups, such as PLWH, MSM, and patients in STIs clinic (29). The results of our study further support these findings, patients who were male, homosexual, and had a history of STIs were at higher risk of syphilis than the other participants. A meta-analysis study found that the overall prevalence of syphilis among HIV-infected patients in China reached 19.9% (29), 7.3% in sub-Saharan Africa (34) and 19.9% in France (35). The 5.6% syphilis infection in our study was lower than the above results, the rationale for this was: our study participants were PLWH with viral suppression, who themselves had higher adherence and lower risk behaviors (36). Syphilis has emerged as a major public health threat, particularly among MSM in China who have a high incidence of syphilis and HIV/syphilis co-infection (33). Traditional healthcare services throughout China include STIs screening for high-risk groups. However, MSM, especially those in rural areas, are difficult to identify and link to care (33). Strengthening case finding and promoting point-of-care testing in order to identify and rapidly diagnose high-risk individuals are important measures that could be taken to address this growing epidemic.

In addition, participants who were treated in municipal and county hospitals had a higher risk of syphilis. Currently in China, over 70% of PLWH are treated in hospitals (25). This decentralization of care has been successful in initiating a larger number of PLWH receiving ART, improving treatment efficacy, and reducing mortality (37). However, in this study, the prevalence of syphilis infection among virally suppressed PLWH was two twice as high in patients receiving care at a hospital than in those receiving care at a community health service center. Structural barriers, such as fewer STIs clinics and a lack of appropriate training in hospitals, may explain this discrepancy (38). Local community health service centers in China have more experience in providing interventions for high-risk sexual behaviors, whereas hospital staff typically focus on HIV treatment and care, without the necessary training to address high-risk sexual behaviors (39). Additional training of hospital staff to identify patients who may be at increased risk, and promotion of routine testing at local clinics may help identify at-risk individuals and reduce on-going syphilis transmission. It may be worthwhile for CDCs and hospitals to coordinate care to take advantage of their respective strengths in the preventing and treating of HIV infection and other STIs in China.

Notably, our findings also showed that the duration of ART did not affect the prevalence of current syphilis infection, suggesting that sexual risk behaviors did not change in this study population regardless of ART status. Among participants who had been on ART the longest (for ≥10 years), the prevalence of syphilis was 4.8%. The impact of ART on risk aversion has been hotly debated. A previous study in Uganda found that high-risk sexual behavior increased among HIV-infected individuals after ART initiation (40). However, other studies have found that risky sexual behaviors declined after years of ART (41). It is of concern that continued risky sexual behavior may increase the transmission of drug-resistant HIV and STIs (42). Therefore, virally suppressed patients on long-term treatment who engage in high-risk behaviors, particularly in the presence of STIs, should be continuously monitored for intervention purposes. Viral load testing at 6 or 9 months after ART initiation is warranted, particularly in patients co-infected with other STIs.

Low education was a risk factor for syphilis infection according to previous studies (43). In contrast to the previous studies, our results showed that a high level of education was a risk factor for syphilis infection. The possible reasons were: first the participants in this study are HIV-infected patients who had HIV virally suppressed and had good treatment compliance. People with low levels of education tend to have poor adherence to treatment, resulting in unsuppressed HIV viral load (36). Therefore, they were not included in this study. Second, in the implementation of U=U strategy, study had shown that highly educated people have higher awareness of U=U strategy (44), PLWH with HIV virally suppressed effectively no risk of transmitting HIV sexually, and therefore might cause them not to use safe behaviors and increased high-risk sexual behavior (45).

This study reports the prevalence of syphilis infection in a cohort of virally suppressed PLWH in eastern China. However, this was a cross-sectional study; thus we could not determine the causal relationship between current syphilis infection and long-term treatment for VL suppression. Further longitudinal cohort studies are needed to assess the incidence of current syphilis among virally suppressed throughout China. Additionally, participants' VL levels were obtained from the 2022 national registry data. VL and current syphilis testing were not performed during the same time period, which may have confounded the results. However, once yearly VL testing is the standard practice in China and in other parts of the world; therefore, all PLWH would receive their VL results according to this schedule. The high response rate and large sample size in this study in a unique subset of PLWH highlights the need for on-going risk reduction among virally suppressed individuals.

## Conclusion

The prevalence of current syphilis infection is high among virally suppressed PLWH in eastern China, and ART duration did not reduce the prevalence of current syphilis infection. On-going sexual health education, rapid identification and diagnosis of at-risk individuals using point-of-care testing, and additional training of health care providers at the provincial and local levels, are warranted to maintain viral suppression and reduce on-going STIs and HIV transmission.

## Data availability statement

The data analyzed in this study is subject to the following licenses/restrictions: the raw data will be provided upon request by XP, xhpan@cdc.zj.cn. Requests to access these datasets should be directed to xhpan@cdc.zj.cn.

## Ethics statement

The studies involving humans were approved by the Institutional Review Board of the Zhejiang Provincial Center for Disease Control and Prevention. The studies were conducted in accordance with the local legislation and institutional requirements. Written informed consent for participation in this study was provided by the participants' legal guardians/next of kin.

## Author contributions

LH: Writing – review & editing, Writing – original draft. XP: Writing – review & editing. JY: Writing – review & editing, Investigation. JZ: Writing – review & editing, Investigation. ML: Writing – review & editing, Investigation. WC: Writing – review & editing, Investigation. CC: Writing – review & editing, Investigation.
